# Social aspects of privacy in technologically assisted dementia care

**DOI:** 10.1007/s11019-025-10317-z

**Published:** 2025-12-19

**Authors:** Eike Buhr

**Affiliations:** Ethics in Medicine, Carl von Ossietzky Universität Oldenburg, Oldenburg, Germany

**Keywords:** Privacy, Dementia care, Technical assistance systems, Ethics, Qualitative research

## Abstract

Technical assistance systems (TA) are increasingly used in the care of people with dementia (PwD). A central ethical concern in this context is the potential violation of privacy. However, the prevailing debate – shaped largely by liberal traditions – tends to conceptualize privacy in terms of individual autonomy. Yet PwD progressively lose their capacity for autonomous decision-making, and their everyday lives are shaped by close caregiving relationships. This study adopts an empirically informed medical ethics approach to examine the value of privacy for PwD and the role of interpersonal relationships in this context. Drawing on 12 interviews with PwD and 15 with caregiving relatives, the findings show that privacy remains a meaningful value for PwD, independently of individual autonomy, and is closely tied to their well-being. Moreover, privacy and relationships emerge as mutually constitutive: on the one hand, privacy supports the maintenance of meaningful relationships; on the other, close relationships can enable PwD to experience and preserve privacy. These insights carry concrete ethical implications for the design and implementation of TA systems, as well as for the medical care of PwD.

## Introduction

Technical assistance systems are increasingly being used in the care of people with dementia (PwD) (Schweda et al. 2019). These include, for example, GPS-tracking systems to help with wandering, systems that assist with activities of daily living, e.g. sensor-based dressing aids that guide or support PwD in performing step-by-step dressing tasks, or monitoring systems to detect falls (Ienca et al. 2017). These assistance systems are also discussed with reference to their implications for close care relationships and for privacy (Buhr and Schweda 2022; Ienca et al. 2018). However, some argue that the specific value of privacy for PwD is not sufficiently addressed (Buhr and Schweda 2025; O’Brolcháin and Gordijn 2019). This may stem from the liberal tradition of the concept of privacy, where a right to privacy was originally conceived as an individual right of defense against state and social intervention (Locke 1988; Mill 2011). Even in more recent conceptions, privacy appears primarily as a right that either presupposes or is functionally oriented towards individual autonomy (Rössler 2005).

Such a narrowly individualistic conceptualization does not consider the perspectives of PwD, as they increasingly lose their capacity for individual autonomy and are increasingly dependent on close care relationships as the disease progresses (Buhr and Schweda 2022). However, PwD and their family caregivers explicitly name privacy as an important aspect of their quality of life (Dichter and Schmidhuber 2016; Mulvenna et al. 2017). This implies that any concept aiming to make the value of privacy plausible for PwD must disentangle the notion of privacy from a strong emphasis on individual autonomy. Rather than focusing exclusively on the individual, it should instead adopt a relational perspective on privacy that considers the lived experience of PwD.

In fact, there are approaches that examine privacy in terms of its social and relational dimension, for example by discussing the significance of privacy for democratic societies (Mokrosinska 2018; Rössler 2015; Seubert and Becker 2019), for (intimate) relationships, or by criticizing the individualistic focus of conventional approaches to privacy (Cohen 2002; Fried 1968). For instance, communitarian theorists do not conceive privacy as an individual right but rather as the protection of practices that are relevant to communities and require privacy (Etzioni 1999). However, the point of reference either remains an autonomous individual, or the focus of privacy is on communal practices rather than privacy for or in concrete relationships. Thus, these accounts consider privacy in terms of its value for societies or in terms of its political or communal dimension. Even though these aspects may also apply to PwD, they still cannot address the fact that the value of privacy for PwD is not primarily functionally oriented towards autonomy or practices that are relevant for society. While some accounts emphasize the importance of privacy for relationships, they disregard the importance of relationships for privacy. This means that one crucial aspect of the *lifeworld* (Husserl) of PwD, namely that relationships can be not only the object but also the subject of privacy, is not considered. Conventionally, relationships appear as the object of privacy: for example, privacy protects personal conversations and shared knowledge from outside intrusion, it shields the domestic sphere in which relationships are lived, and it secures the freedom to make choices about relationships themselves, such as with whom one wishes to live or maintain close contact. By contrast, understanding relationships as the subject of privacy highlights that privacy can itself be constituted through relationships. For example, a close relative may manage personal information, co-decide on matters of care, or provide the conditions necessary for PwD to remain at home.

This article addresses this conceptual gap by exploring the role of relational aspects in analyzing the value of privacy in dementia care. The focus lies on the question of how close caregiving relationships provide a foundation for understanding the value of privacy for PwD. To tackle this question, in addition to a conceptual-theoretical analysis of privacy, an empirical exploration regarding the social dimension of privacy is required. Thus, I conducted and analyzed 12 interviews with PwD and 15 interviews with caregiving relatives to explore the extent to which social aspects play a role regarding the value of privacy in the technically assisted care of PwD.

This paper addresses two key objectives. First, it explores the social dimension of privacy, examining its significance and value for PwD. Second, it analyzes the role of privacy in the context of technically assisted dementia care. In doing so, a more precise understanding of how technical assistance systems affect their care can be achieved. This requires a comprehensive analysis of these systems’ implications not only for privacy but also for interpersonal relationships, and a conceptual link between these two factors. Through this exploration, the nuanced effects of technology on both aspects of care can be better understood, highlighting the fundamental importance of close and familiar relationships for the well-being of those affected (Smebye and Kirkevold 2013).

I proceed as follows: Sect. 2 contains a brief description of the concept of privacy with a particular focus on its relational or social aspects. The section concludes that conventional concepts of privacy only inadequately represent the value of privacy for PwD and that the conceptual-theoretical analysis of the concept of privacy has reached an impasse. In Sect. 3, the method of the interview study is explained. Section 4 presents the results of the interviews and analyzes them in the context of the debate on the social dimension of privacy. Here, I show that participants understand privacy as a fundamentally social phenomenon that is not adequately captured by existing concepts. Section 5 provides a discussion of the results and an outlook on their implications as well as a way forward. It becomes evident that technical assistance systems, insofar as they aim to foster privacy through individual independence, fall short by overlooking the enabling role of relationships in the perception and experience of privacy.

## Background: privacy and its social aspects

To explore the value of privacy for PwD and the role of relationships in this context, it is first necessary to engage with conceptualizations of privacy. Despite the efforts of many scholars to define the exact nature of privacy and its distinct value, there is no consensus regarding its specific essence. Rather, privacy is a “concept in disarray” (Solove 2009, p. 9). The concept covers different notions commonly associated with privacy, e.g., “freedom of thought, control over one’s body, solitude in one’s home, control over information about oneself, freedom from surveillance, protection of one’s reputation, and protection from searches and interrogations.” (Solove 2009, p. 1088).

Against this backdrop, Rössler (2005) distinguishes three dimensions of privacy, i.e. local, decisional, and informational privacy. These dimensions form the core of her account. For Rössler, all three dimensions concern different forms of control over access: control over access to places (local privacy), control over access to one’s decisions and personal life (decisional privacy), and control over access to personal information (informational privacy). In Rössler’s account, local privacy refers to the ability to exclude others from physical spaces in which one withdraws from public view. She conceptualizes this dimension as layered, ranging from the outer sphere of one’s property to the most intimate layer of one’s own body. Other authors have similarly highlighted its protective function and its relevance in medical contexts, especially in terms of bodily privacy (Allen 2014). Constitutional guarantees of the home underscore the significance attributed to this dimension (Koops et al. 2017; Ch. 4.3). Decisional privacy for Rössler concerns the authority to determine who may interfere with or influence decisions about one’s personal life (Rössler 2005). The prominence of this dimension is reflected, for instance, in US jurisprudence, where decisional autonomy has been framed as “constitutional privacy” (Griswold v. Connecticut, 381 U.S. 479 (1965); Roe v. Wade, 410 U.S. 113, (1973); DeCew 1997, p. 24 f.). Finally, informational privacy in Rössler’s account refers to control over access to personal information. Her view aligns with earlier control-based conceptions that emphasize restricted access to such information (Schoeman 1984; Westin 1967). This control enables individuals to manage how aspects of themselves are presented in social contexts, including the shaping of public identity. It is noticeable that in Rössler’s as well as in most other conceptions, the value of privacy is generally either functionally oriented towards autonomy or presupposes a certain degree of autonomy and appears above all to be individually valuable. In the following analysis, these dimensions should not be understood as rigid categories but rather as a heuristic framework that helps to clarify the different aspects of privacy.

In addition to conceptions that place the value of privacy for autonomous individuals at the center of their analysis, other authors emphasize the social significance of privacy and its function for groups, relationships, or societies. Fried (1970) and others point out that information serves as the currency of social relationships and that the regulation of its exchange plays a decisive role in determining the degree of intimacy within relationships. They argue that (informational) privacy enables certain relationships in the first place (Cohen 2002; Rachels 1975; Roessler and Mokrosinska 2013). Moreover, communitarian approaches criticize the individualistic focus of liberal approaches to privacy. Communitarian theorists do not conceive privacy as an individual right but rather as the protection of practices that require privacy and are relevant to the community (Etzioni 1999). Thus, for advocates of communitarian approaches, individuals do not have a right to privacy based on their status as autonomous individuals, but as members of a specific community. Other studies focus on the political and social dimensions of privacy in their analysis and emphasize the democracy-promoting character of privacy. Following Gavison (1980), Solove (2009) argues that privacy supports and promotes the moral autonomy of responsible citizens in democratic societies. Thus, from the point of view of democracy theory he supplements the individual value of privacy by a political function (Stahl 2020). Against the backdrop of modern information technology and its ability to target not only individuals but also groups, Floridi (2017) and Taylor (2017) argue for a right to privacy for groups. The social dimension of privacy therefore emphasizes the aspect that privacy is not only of value for individuals but is also relevant for groups and enables or protects relationships.

While these conceptions are not narrowly individualistic, they are functionally oriented towards (moral) autonomy or presuppose an autonomous individual as a politically active citizen. They therefore overlook that privacy not only serves to protect certain relationships but conversely that relationships can also serve to enable privacy in the first place. This is particularly relevant for PwD, who are increasingly dependent on close care relationships. The progressive impairment of cognitive abilities in dementia affects executive functions, leading to a loss of practical everyday skills and limiting the ability to process more complex information or make well-considered, self-determined decisions. This can also lead to self-harm or behavior that endangers others, which in turn requires an increasing reliance on caregivers. This particular importance of relationships for PwD is also reflected in relevant standards and guidelines for nursing practice, which emphasize that professional care for PwD should take place in relationships that enable all those involved to experience security, fulfilment, and a sense of purpose (Wilson et al. 2009). In this context, the fundamental importance of close and familiar relationships for the self-determination and well-being of the person receiving care is frequently emphasized (Nolan et al. 2004).

Against this backdrop, the theoretical study of the concept of privacy has reached an impasse, as no existing concept can represent the specific value of privacy that arises in the context of the social constitution and dependence on close care relationships of PwD, especially in the context of technical-assisted care. Approaches that address privacy in the context of information technologies primarily focus on surveillance technologies that monitor autonomous citizens and risk infringing upon the exercise of fundamental democratic rights (Taylor et al. 2016). Consequently, the living conditions and perspectives of PwD are largely overlooked in discussions on privacy in general as well as in the specific context of technology-assisted dementia care.

## Methods

This study pursues an empirically informed approach (Ives et al. 2018). To explore how PwD experience privacy, what value privacy holds for them in different contexts, and what role (close care) relationships play here, I conducted semi-structured interviews with PwD and their family caregivers. By empirically exploring possible dimensions of privacy, the specific perspective of PwD can be incorporated into the ethical discourse on the value and function of privacy in technically assisted dementia care. Such an exploration can reveal aspects of privacy that have been overlooked in discussions on the social dimension of privacy so far.

A total of 27 interviews with PwD (*n* = 12) and their family caregivers (*n* = 15) were analyzed. The interviewees were recruited via local self-help groups and care homes in Oldenburg and Bremen, via the REHA Center Oldenburg and via the German Center for Neurodegenerative Diseases (DZNE), Rostock as a project partner in the project EIDEC.^1^ Potential participants were included in the study if they had a diagnosis of dementia or a score of less than 25 on the Mini-Mental Status Test (Mendez 2022). Family caregivers were included if they were or had been a caregiver in the close environment of a PwD.

The attitudes and views of PwD and their family caregivers regarding privacy were explored using a semi-structured guide. The guideline contained questions about everyday life, the living and care situation of the interviewees, and their experiences with health-related technical aids. Semi-structured interview guidelines were chosen to ensure that relevant thematic areas of the EIDEC project were addressed, while still allowing participants to articulate their individual perspectives. This approach enabled comparability across interviews while retaining the flexibility to capture nuanced views on privacy.

The interviews focused on respondents’ attitudes towards four different technical assistance systems: a GPS tracker, a dressing aid in two different versions, and an emotion recognition system to detect challenging behavior at an early stage. As these systems collect sensitive data, determine the location of the user, are used in the user’s own private home, intervene in everyday activities, or aim to change behavior, the use of these systems potentially violates the informational, local, and decisional dimension of privacy. These systems were selected because they represent typical applications of technical assistance technologies in dementia care, most prominently robotic systems, smart-home technologies, and wearables (Astell and Semple 2019). In addition, they reflect a range of common care-related tasks, including orientation, dressing and the management of challenging behavior (Schweda et al. 2019).

As most of the participants were only familiar with a GPS tracker and had little to no experience with technical assistance systems, the presentation of the techniques was accompanied by a standardized set of short written descriptions and explanatory illustrations to facilitate understanding on part of the interviewees. The GPS tracker was presented as a wearable location-tracking device; the dressing aid was shown in two versions, one depicting a sensor-based prompting system and the other an assistive robotic device providing physical support during dressing. Finally, the emotion-recognition system was presented as a camera-based tool intended to detect early signs of challenging behavior. The interviewer first briefly described the purpose and mode of operation of each device, then showed the accompanying illustration and finally invited participants to discuss their attitudes, potential use and perceived implications for privacy.

The interviews were structured around the four introduced technologies. Within these sections, participants were asked regarding their basic attitude towards the technology and their willingness to use the respective devices. Moreover, the guideline contained open-ended questions regarding the possible influence of the assistance systems on their independence, safety, privacy, quality of life, and the relationship with their respective caregivers. The privacy dimension was introduced with the question “How does using this technology affect your privacy?” for PwD, and “In what ways do you think this technology could affect the user’s privacy?” for caregiving relatives. Because the interviews were structured around each technology rather than by topic blocks, discussions on privacy did not occur in a temporally distinct segment.

The interviews took place from October 2020 to December 2021 and were conducted in the respective care facilities, at the DZNE or in the private homes of the participants. The duration of the interviews varied between 26 and 97 min, with an average duration of 43 min. Because the interviews were structured around each technology rather than by topic blocks, discussions on privacy did not occur in a temporally distinct segment. The length and depth of the privacy-related discussion therefore varied substantially across interviewees, depending on which aspects they considered most salient. The age of the participants ranged from 37 to 92 years, and the level of education ranged from secondary school qualifications to university degrees. Four PwD lived in a nursing home and one family caregiver lived in an assisted living facility. The remaining participants lived with their partners or family members in their own home. Ethics approval was obtained from the Ethics Committee of the Medical Faculty of the University of Oldenburg prior to the interviews.

The participants’ statements were recorded, transcribed and analyzed using ATLAS.ti^®^. A coding guideline was developed for the analysis. In the coding guideline, the informational, local and decisional dimensions of privacy served as deductive categories. These categories functioned as sensitizing concepts during coding not as predetermined interview questions. Social aspects of privacy were added inductively in response to their frequent appearance in the data and the material was analyzed following Kuckartz’ method of qualitative content analysis. For the systematic identification and subsequent analysis of patterns in participants’ accounts regarding the value of privacy, qualitative thematic analysis following Kuckartz and Rädiker (2023) was applied. In this analytic approach, categories are generated and refined during the coding process rather than during the interviews themselves; accordingly, the identification of privacy dimensions occurred during data analysis and not during the interviews.

## Results: the social dimension of privacy

Many participants emphasize the specific relational character of privacy and its value in the context of dementia care. It is striking that the interviewees emphasize well-being as a function of privacy and regard relationships not only as the object of privacy, i.e., what privacy protects, but also as its subject, that is, the means through which privacy is constituted and maintained. The participants’ statements are categorized according to the local, decisional, and informational dimensions of privacy. These dimensions, however, are not always sharply distinct and may overlap. For this reason, they are best understood not as rigid classifications but as a heuristic framework that both structures the categorization of statements and facilitates a systematic discussion of the social aspects of privacy.

### Social dimension of local privacy

Regarding the social dimension of local privacy, two distinct themes emerge from the interviews. One addresses the importance of local privacy for the perception and maintenance of social relationships and sees this, in turn, as a fundamental aspect of well-being. The second theme focuses on the meaning and normative implications of sharing a private living environment within relationships. This illustrates that relationships can also be a prerequisite for perceiving aspects of local privacy. In this respect, relationships are the subject of local privacy. The distinction lies in whether local privacy is treated as a spatial condition that enables social relationships, or as a relationally constituted sphere that exists only through shared caregiving arrangements. In the following, I refer to these as (1) local privacy as a condition for maintaining social relationships, and (2) local privacy as constituted through shared living arrangements.

Ms. Werner, an 82-year-old caregiver for her husband with advanced dementia, describes the importance of her own living environment for her relative and how it is only there that he is able to maintain social contacts. Her account exemplifies the first theme, namely local privacy as a precondition for maintaining social relationships and supporting well-being.

Ms. Werner “Contacts […] are also important for him. So visitors. Even if he can no longer categorize them. […] What do you call that? A kind of sociability too, right? […] [A]lthough increasingly only at home. Because he can no longer find his way around. When we’re somewhere else, he’s afraid we won’t find our way home.” (Caring relative, 82).

This interview passage highlights the role and significance that Ms. Werner’s spouse's home holds for him. Here, his own private home, as one aspect of local privacy that he is familiar with and provides him with a sense of security, serves as a condition for the possibility of maintaining social contacts. The statement underlines the importance of local privacy for PwD, as it serves as a condition for being able to maintain social contacts, which is directly beneficial to the well-being of the PwD. Analogous to existing concepts of the social dimension of privacy, the focus here is on the importance of privacy *for* relationships. This not only includes the possibility of exchanging information but also requires a private space that is necessary for the perception of intimate relationships. Accordingly, it is necessary to consider how technical assistance systems shape local privacy by mediating the relational dynamics of the home. For instance, when video telephony replaces personal visits or when visitors feel discouraged from entering because the technology makes them feel monitored.

Remaining in their own home plays a key role for PwD (Fæø et al. 2019). Staying at home not only enables social relationships but relationships are sometimes also necessary to ensure that people can remain in their own home and thus maintain their local privacy, as it is the home that provides a protected space and the possibility for spatial seclusion and control over contact with others. This is illustrated in the following quote by Ms. Schneider. Here, she emphasizes what it means for her relationship with her husband to live together. She directly conceives local privacy as social, i.e. as a shared sphere of privacy. The following case illustrates the second theme, in which local privacy is constituted through shared living arrangements and caregiving responsibilities.

Author “To what extent do you think it would make a difference if you [using a GPS tracker, Author] could see where your husband is, or a care service or a doctor?”

Ms. Schneider “It depends on the condition, on the overall appearance. At the moment it would be important for me [to be able to locate him with a GPS tracking device, Author]. But as far as I know, this is a progressive process. And there are other things to come. […] But as long as he’s at home, I feel responsible for him and then I would definitely like to be involved in such things.” (Caring relative, 73).

Ms. Schneider discusses the question of whether it would make a relevant difference to her if she were personally able to track her husband’s location via a GPS device, as opposed to medical staff having that capability. While the question is intended to elicit whether she would have concerns about informational privacy in the event that such sensitive data were shared with third parties, Ms. Schneider instead responds by highlighting the special significance that sharing a private home holds for her caregiving relationship with her husband. Her response illustrates the extent to which couple relationships also create responsibility. Ms. Schneider states that she feels responsible for her husband “as long as he is at home”. In this way, the responsibility that results from a shared private home and in this case, a shared living arrangement, takes on a special form of urgency, directness, and immediacy. Moreover, the quote also illustrates the extent to which a close care relationship – here supported using a GPS tracker – enables Ms. Schneider’s husband to remain in his own home. In this regard, relationships function as the subject of local privacy, as it is the relationship that enables Mr. Schneider to remain in his familiar home, which offers a protected space and allows for spatial seclusion.

### Social dimension of decisional privacy

Participants also discuss social aspects of decisional privacy. The quoted statements can be differentiated based on the extent to which they conceptualize relationships as either the object or the subject of this dimension of privacy. While one quoted participant emphasizes the possibility to determine the number and intensity of social contacts without having to give other people a say in the matter, the other theme concerns the desire to grant others a say in specific matters, i.e. include others in decision-making. Here, decisional privacy includes the right to deny others a say, but also to grant it. These aspects are discussed under decisional privacy, as the focus here lies on the question whom to deny or grant a say regarding decisions concerning one’s private conduct of life.

In the following quote, Ms. Braun describes her relationship with the other residents of the care facility she lives in and how this relationship has changed over time. Although Ms. Braun does not explicitly refer to a specific technology in this passage, her reflections occurred within a discussion about the potential introduction of assistive technologies. They illustrate how decisions concerning such technologies are embedded in broader patterns of regulating social contact. This example therefore highlights the first aspect of the social dimension of decisional privacy: the ability to regulate social contacts independently.

Ms. Braun “And I’m happy when I’m alone. […] So there are such and such. And I feel most comfortable when I’m alone, when I can decide for myself. […] [W]e were such a small circle, here in the house, with whom I had good contact […] and also talked a lot […]. But one day it was too much for me. […] I […] like to be alone. But I also like being in large groups. It’s just that I want to decide for myself. I don’t want to be squeezed in somewhere. (Person with dementia, 81)

Ms. Braun stresses her need for seclusion and the opportunity to be alone. It is not foremost the seclusion that seems to be a part of her well-being but above all the possibility of being able to determine this herself. Here, she claims to decide for herself whether and when she wants to engage with the other residents, highlighting the importance of being able to decide such private matters independently. By emphasizing that she does not want to grant anyone a say in this, social contacts appear as an object of decisional privacy in her considerations. This means, that decisional privacy secures the ability to make choices about relationships themselves. Thus, Ms. Braun’s statement indicates that the relational dimension of decisional privacy is a matter of regulating communication within social contacts as well as regulating social contacts as such.

In the following statement, Ms. Schmidt reflects on the acquisition of an assistance robot, explicitly involving her daughter in the decision-making process. The next quote shows the second aspect: the willingness to grant others a say in specific decisions.

Author “What concerns would you have about a robot running around the apartment?”

Ms. Schmidt “I would always mess with him. And complain. […] I would say, shitty thing. You’re in my way. I can’t even get past you with my walker. […] Nope. I don’t think I need it. And if I did, I don’t hope that I’ll get old enough to need it. I really hope so. And if then you [addressing the daughter, Author] know, then we would have to get one of those things.” (Person with dementia, 84).

After this spontaneous rejection, Ms. Schmidt concedes that nursing and medical necessities might also play a role, acknowledging that she cannot fully assess the scope of such a decision. Strikingly, she then turns to her daughter granting her the right to co-decide. This gesture expresses trust and illustrates how shared decision-making with close caregivers can be experienced as relieving. Whereas conventional accounts of decisional privacy describe a sphere of personal choice shielded from third-party interference, Ms. Schmidt points to the complementary possibility of actively including trusted others. In this sense, her relationship with her daughter becomes the subject of decisional privacy: it enables her to preserve privacy by involving a close relative rather than leaving the matter to unknown third parties. This extends conventional notions of social privacy, where relationships are seen only as its object. The statement demonstrates, first, that relationships provide the very condition for experiencing decisional privacy and second, that close relationships are of particular importance for PwD. Against this backdrop, it is crucial to examine how technical assistance systems may affect intimate caregiving relationships and thereby shape decision-making processes.

### Social dimension of informational privacy

Participants also referred to social aspects of informational privacy in their statements. For example, in the statement quoted the collection of (health-related) data is not considered as an intrusion into informational privacy if only close family members have access to this data.

In the following quote, Mr. Krüger, a 79-year-old man with incipient dementia, distinguishes between authorized and unauthorized access on the basis of intention which in his view is shaped by the underlying social relationship. He discusses this while evaluating an assistance system for emotion recognition. The following example demonstrates how informational privacy is evaluated through the lens of the underlying relationship.

Mr. Krüger “Well, when something like that is done, you probably try to teach the person that it’s not monitoring, but a support facility. Yes, that’s important, isn’t it? [I]n prison you’re under surveillance, I say, right? And in the family, you’re watched so that you can help. That’s how I see the difference now. (Person with dementia, 79)

Mr. Krüger contrasts surveillance in prison with monitoring in a family context which he interprets as supportive rather than intrusive. His reasoning highlights the relational character of informational privacy: health-related information is seen as shared information within a relationship. Accordingly, sharing information in such contexts is not automatically perceived as a violation of privacy since the relationship itself constitutes a private sphere. This perspective has normative implications for the implementation of technical assistance systems as the evaluation of data collection depends on where, by whom and with what intention information is gathered. Informational privacy is thus conceived as inherently relational, with the depth and quality of the relationship shaping how privacy boundaries are drawn and when their transgression is experienced as intrusive. For example, monitoring by a caring relative may be regarded as acceptable support rather than an invasion of privacy.

## Discussion

This paper examines how PwD experience privacy in the context of technical-assistance systems, the value they attribute to it, and the role of close caregiving relationships.

With respect to local privacy, two perspectives emerged from the participants’ statements. One highlights the home as a familiar environment that enables PwD, even in advanced stages, to sustain social relationships. The other concerns the responsibilities that arise within shared living arrangements in close caregiving relationships. In this second view, the provision of care within the home becomes the very condition that allows the PwD to remain in this environment and thereby to maintain local privacy.

The special meaning that one’s own home and associated aspects have for PwD has been addressed in various places (Dekkers 2009, 2011; Fæø et al. 2019). For instance, Dekkers distinguishes between four possible meanings of the term, such as one’s own home, one’s own body, one’s psychosocial environment, and a spiritual dimension (Dekkers 2009). In the context of dementia, the concept of home may serve to “maintain […] a sense of personal identity and independence” (Dekkers 2011, p. 298). This is of particular relevance here since older people in general rely on their home more than younger people, and PwD in particular often wish to remain in their private living environment for as long as possible (Krasner 2005). As relationships play an extraordinary role (Buhr and Schweda 2022; Krasner 2005) – one that is directly tied to their individual well-being (Smebye and Kirkevold 2013) – local privacy should also be analyzed with regard to its social dimension. This is especially relevant because the interviews suggest that relationships may serve as a prerequisite for PwD to experience local privacy. This extends existing literature, which typically frames privacy as a precondition for relationships, by demonstrating that relationships can equally function as a precondition for privacy.

Decisional privacy is often overlooked in dementia research due to its close association with autonomy (O’Brolcháin and Gordijn 2019). Yet the interviews revealed that PwD still claim a personal sphere in which they exercise decision-making authority. A first theme highlights the importance of being able to withdraw to a familiar and private space for sustaining intimate relationships. While this perspective on privacy is similar to Julie Cohen’s account on relational privacy regarding the possibility of regulating communication within social contacts (Cohen 2002), the analysis further shows that regulating social contacts as such is regarded as one’s private affair. Here, relationships appear as the object of decisional privacy. This means, that the way one wishes to conduct their relationships is object to their private decisions.

A second theme concerns the wish to include trusted others in decisions affecting everyday life. The social dimension of decisional privacy not only describes the right to deny third parties the right to have a say but also to grant it to them and above all to determine for oneself to whom this right should be granted. Here, relationships are the subject of decisional privacy since it is the relationship itself that enables PwD to keep some degree of decision-making authority. While most accounts of decisional privacy mainly focus on physical and sexual self-determination as well as aspects of identity formation (Allen 2014), the analysis here suggests a conception of relational privacy that focuses on relationships themselves. This underrepresented aspect of decisional privacy seems to be an important norm especially for people in nursing homes as regulating social contacts is often insufficiently provided for (Abbott et al. 2017). This highlights that the value of privacy should not be understood solely as a means of preserving autonomy, but also as an essential aspect of individual well-being.

A central aspect of the social dimension of informational privacy is that it is conceived within relationships. The quoted statement indicates that informational privacy is constituted through the relational contexts in which individuals live and its meaning depends on how sensitive information is shared and handled within these contexts. This perspective highlights first that relationships themselves can be the subject of informational privacy, enabling or shaping its realization, and second that the depth and quality of a relationship determine how boundaries of informational privacy are interpreted and when a transgression is experienced as a violation. Such an understanding of informational privacy corresponds to the approach of Nissenbaum (2010), for whom informational privacy consists in a “right to *appropriate* flow of personal information” (p. 127) and who explicitly emphasizes the role of the actors and the social context in which information circulates (140 f.).

The statements regarding social aspects of privacy represent a decisive extension to conventional concepts of privacy in which relationships are solely considered as the object of privacy, focussing on privacy as a condition of relationships or the right to withdraw from them. Rather, the view proposed here is conceptually close to the concept of relational autonomy. Mackenzie and Stoljar (2000, 2023) argue for a concept of autonomy that acknowledges the social constitution of autonomy and emphasizes that people always act within social relationships where external influences on the formation of will do not necessarily undermine individual autonomy. This emphasis on the social constitution of persons applies even more in the context of close (dementia) care relationships. This notion of relational autonomy can also be extended to social aspects of privacy where co-deciding does not infringe on decisional privacy, reliance on others to remain at home does not diminish local privacy, and sharing information with loved ones is not a violation of informational privacy. Rather, close relationships enable PwD to maintain and experience privacy. In this regard, relational aspects cut across all three dimensions of privacy and highlight how such relationships in dementia care actively constitute the conditions under which privacy can be sustained.

Against the backdrop of the importance of relationships for the care of PwD and the conceptual connection with questions of privacy, the considerations made here are particularly relevant for the design of assistive technologies in dementia care where violations of privacy represent a pressing ethical concern which is not sufficiently addressed in the ethical debate (Ienca et al. 2018). First, relationships should not be seen merely as the object but also as the subject of privacy. This means that relationships should not be analyzed primarily in terms of their potential privacy violation but also regarding their privacy-enabling function. Secondly, privacy is not only relevant in terms of autonomy or valued for its autonomy-enabling function. Rather, alternative resources such as well-being can also serve as a basis for a right to privacy (Buhr and Schweda 2025). Accordingly, the results reflect an expanded understanding of the social dimension of privacy underscoring the need for greater emphasis on relationships when evaluating the impact of technical assistance systems on the privacy of PwD.

This means that on the one hand technical assistance systems should be evaluated in terms of the extent to which they enable potential users to remain in their own homes for longer – an essential aspect of maintaining local privacy (Novitzky et al. 2015). It is therefore important to examine how such systems affect caregiving relationships shaped by shared living arrangements. The analysis suggests that certain technologies such as GPS tracking may alleviate caregiver burden and thereby support the wish to remain at home. On the other hand, it is necessary to consider to what extent the use of technical assistance systems changes the perception of one’s own living space to such an extent that it is no longer considered a home (Welsch and Buhr 2022). The challenge is not simply to make systems invisible which risks deceiving users but to integrate them meaningfully into the living environment. The quoted statements indicate that this is less a matter of aesthetics than of subjective well-being. A further question is whether the presence of such technologies affects the willingness of guests to visit as they may feel monitored or uncomfortable. This concern is particularly relevant for smart-home technologies and emotion-recognition systems that track movement or even facial expressions (Tian et al. 2024).

With respect to decisional privacy it is crucial to ask who decides on the installation of an assistance system and how such technologies influence close caregiving relationships (Buhr and Schweda 2022). It is essential to recognize that PwD still have a need to discuss and reflect on personal lifestyle issues in private, even if they may not fully comprehend the broader implications of these decisions. This underscores the importance of familiar caregiving relationships in facilitating a sense of decisional privacy.

Building on this the evaluation of technical assistance systems must also consider whether they preserve the ability of users to exercise control over their social interactions. For instance, it is crucial to consider whether PwD can still decide when they are available for video calls or whether they experience certain devices – such as the dressing robot discussed in the interviews – as intrusive presences in the home that they cannot refuse. Decisional privacy can further be supported by implementing systems gradually and ensuring that their use remains reversible thereby preventing individuals from feeling bound by irrevocable choices. Moreover, design and deployment should explicitly acknowledge the relational context of decision-making in dementia care enabling shared but transparent arrangements with caregivers.

With regard to informational privacy another theme concerns the notion of shared informational privacy within close relationships. This resonates with Nissenbaum’s concept of contextual integrity. This perspective underscores that data collection is not automatically a violation of privacy; rather, information can be understood within the framework of a relationship and thereby appear as relational information. In this sense, a GPS tracker or an emotion-recognition system may not necessarily undermine privacy but can even constitute an expression of it provided that access to the data remains confined to close relatives. Here, close relationships enable informational privacy instead of posing a threat to it.

In a further step it needs to be determined how individually expressed privacy preferences should be weighed against other values, such as security, when designing the functioning of technical-assistance systems (Buhr et al. 2024).

## Conclusion

This paper is one of the few contributions that explicitly analyzes the concept of privacy in the context of dementia (Buhr and Schweda 2025; O’Brolcháin and Gordijn 2019). To the author’s knowledge, it is also the only work that explicitly incorporates the perspective of PwD on this issue. As existing concepts of the social dimension of privacy are narrowly individualistic or functionally oriented towards autonomy, they cannot capture certain aspects of privacy that arise in the specific living situation of PwD, a situation shaped to a considerable extent by close care relationships. The approach presented here advocates for a strong interpretation of relational privacy – one that not only highlights the role of privacy in supporting relationships but also recognizes the fundamental importance of relationships for enabling the experience of privacy. This perspective is particularly relevant in dementia care, where the maintenance of privacy often depends on trusted caregiving relationships rather than on the individual alone. Recognizing this distinction broadens established accounts of privacy by showing that it is not only an individual right but also a social phenomenon that is sustained or enabled through close relationships. The findings have implications not only for conceptualizing privacy but also for implementing technical assistance systems in dementia care. In close caregiving arrangements, privacy should be understood in its relational dimension, which means that the evaluation of such technologies must consider the extent to which they support or disrupt the relationships that make it possible to experience privacy across its different dimensions.

## Data Availability

The data that support the findings of this study are available from the corresponding author upon reasonable request.

## Footnotes

1 Research Grant: 01GP1901B
